# A retrospective cohort survey of problems related to second childbirths during the 2-child policy period in Jiangbei District of Ningbo City in China

**DOI:** 10.1097/MD.0000000000010604

**Published:** 2018-05-04

**Authors:** Jun Fu, Yu Qu, Fei Ji, Huijuan Li, Fangyuan Chen

**Affiliations:** Department of Gynecology and Obstetrics, Ningbo Women and Children's Hospital, Ningbo, China.

**Keywords:** 2-child policy, cesarean section, elderly parturient, gestational diabetes mellitus, postpartum hemorrhage

## Abstract

From 1979 to 2014 in China, a 1-child policy was imposed to control population growth. During 2014 to 2015, families in which 1 spouse was only 1 child were eligible to apply for planning a second child. To foresee issues affecting obstetrical departments related to the introduction of the universal 2-child policy in 2016, we retrospectively investigated the demographics and health-related outcomes of second pregnancies in families applying for a second child in Jiangbei District of Ningbo City during January 17, 2014, to January 14, 2016.

A retrospective cohort survey was conducted for Jiangbei District of Ningbo City from January 17, 2014, to January 14, 2016, with reference to data from 2012 to 2014.

Applications for a second birth increased after implementation of the 2-child policy, from 505 in 2012 to 2013, to 1222 in 2014 to 2015. Until the end of this study (December 31, 2016), 739 women gave birth to a second child, among whom 21.38% were aged ≥35 years. Rates of cesarean deliveries (59.68%) and gestational diabetes mellitus (14.21% of women) were each positively associated with the age of the mother. Among women aged ≥35 years, 37.97% refused prenatal screening.

Introduction of the 1-child policy encouraged more families to apply for a second child, with many women aged ≥35 years, leading to higher rates of cesarean deliveries and adverse complications. A high percentage of eligible older women refused prenatal screening. Obstetric departments should adjust perinatal health management schemes to prepare for similar probable changes associated with the universal 2-child policy.

## 1 Introduction

Before implementation of the family planning (1-child) policy of China in 1979, the population increased from 540 million in 1950 to over 800 million in 1970.^[1,2]^ After the introduction of the family planning policy, fewer births and the decrease in population growth led to a demographic dividend and significant economic development. However, the population has aged in the last 3 decades and the demographic dividend is vanishing as death rates rise.^[3,4]^ To improve the demographic balance, in 2014, the Chinese central government relaxed the 1-child policy, allowing couples to have a second child if 1 spouse was only 1 child.^[5]^ In October 2015, a universal 2-child policy was implemented, allowing each couple to have 2 children.^[6]^ For Zhejiang province in China, the 2-child policy was put into effect on January 17, 2014, and was replaced by the universal 2-child policy on January 14, 2016.

Undoubtedly, the universal 2-child policy can optimize the demographic structure, increase the supply of labor, and ease the economic pressure due to the aging population. However, increases in the number of pregnancies among older women will lead to inevitable challenges in obstetrics. The demographic and health effects of this major population reformation must be investigated, to prepare for these and other challenges.

In this study, a retrospective cohort survey was performed in Jiangbei District of Ningbo City to investigate the health-related progress and outcomes of second pregnancies in families who applied for a second child. With this study as a reference, we may determine the demographic, social, and reproductive issues that resulted from ending the 1-child policy and recommend specific measures to overcome the challenges of the universal 2-child policy.

## 2 Methods

The ethics committee of Ningbo Women and Children's Hospital approved this research. Each subject provided written consent.

### 2.1 Subjects

We retrospectively analyzed the health-related progress and outcomes of pregnancies from families eligible to apply for a second child during the 2 years in which the 2-child policy was active, that is, from 2014 to 2016 in Jiangbei District of Ningbo City. Data from before the 2-child policy period were applied as a reference.

### 2.2 Data and information collection

For this study, we collected the data and information through several means. In accordance with the family planning policy regulations, in Jiangbei District of Ningbo City, eligible couples who wished to plan a second pregnancy applied to the local government for permission. The applying couples and justifications for application were published on the website of the Health and Family Planning Bureau of Jiangbei Ningbo (http://www.jbws.gov.cn/). Thus, we collected data regarding reproductive features of families from the bureau website. In addition, we collected medical information and data regarding general features through query of electrical medical records from Hospitals in Jiangbei District of Ningbo City. Finally, additional relevant data were obtained by telephonic follow-up, concerning problems during pregnancy, complications, and terminations of pregnancies. Thus, we collected comprehensive data concerning general demographics, pregnancy planning, and complications and outcomes of pregnancy.

### 2.3 Statistical analysis

Data are shown as mean ± standard deviation, or percentage, as appropriate. Statistical analysis was performed using mathematical software (SPSS version 20.0; SPSS Inc, Chicago, IL). The Chi-squared (χ^2^) and Fisher exact tests were used to analyze discrete variables between groups. *P* < .05 (double-sided) was considered statistically significant.

## 3 Results

### 3.1 General features of planned reproduction in Jiangbei District of Ningbo City

#### 3.1.1 Number of families applying for a second-child pregnancy

**Figure 1:**
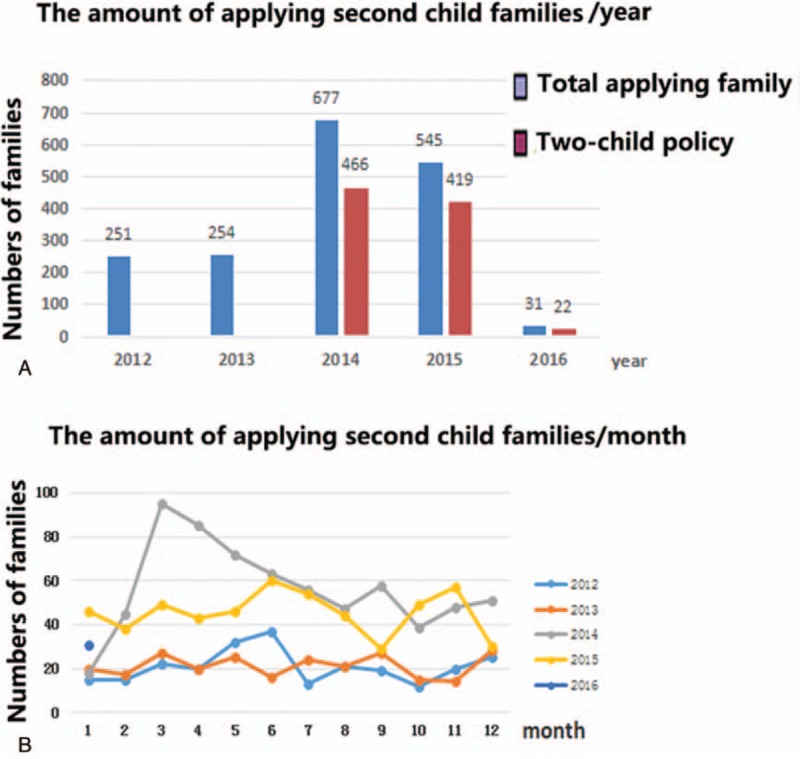
Numbers of families applying for a second child from 2012 to 2016. (A) The total number of annual applications more than double during the years of the 2-child policy (2014–2015). (B) The number of applications rose rapidly in the initial months of the 2-child policy in 2014, and then gradually decreased and stabilized at a level that remained higher through 2015, compared with previous years.

At the end of 2013, there were 101,013 families, with a registered population of 241,802. Before implementation of the 2-child policy, during the years 2012 and 2013, the number of families applying for a second child was 251 and 254, respectively (Fig. 1A). After the 2-child policy was introduced in 2014, couples who were eligible to apply for a second child included those for whom at least 1 spouse was the only child of his/her parents. During the first year (2014), the total number of families applying for a second child was 677. In 2015, the total number of families applying for a second child slightly decreased (545) but remained high.

During the 2 years of the 2-child policy (January 17, 2014–January 14, 2016), 907 families applied for a second child under the new terms, or 466 in 2014, 419 in 2015, and 22 in 2016, accounting for 72.39% (907/1253) of the total number of applications.

We further analyzed the monthly number of families applying for a second child for the years from 2012 to 2015 (not including data in 2016 due to a small size of samples) (Fig. 1B). During 2014 and 2015, in most months, the number of applying families exceeded the number applying in the same month in 2012 and 2013. The number of applications increased sharply during the first months of the 2-child policy but dropped and stabilized thereafter.

#### 3.1.2 Reasons for planning a second child

**Figure 2:**
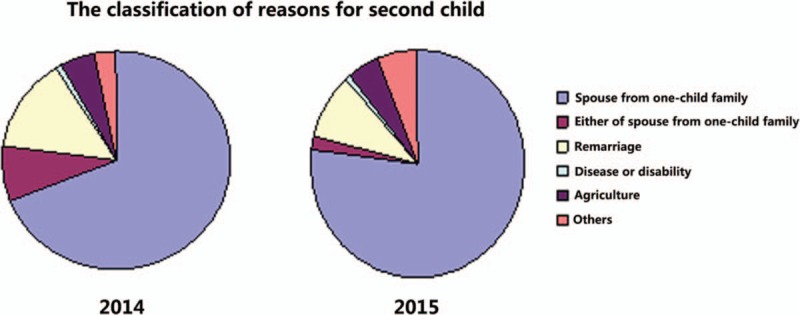
Justifications for applying for a second child during the years of the 2-child policy. In both 2014 and 2015, the predominant justification was that at least 1 spouse was an only child; the second major justification was remarriage.

After implementation of the 2-child policy in 2014, many families were eligible to have a second child and the number of families who applied increased markedly (Fig. 2). The major justification for applying in 2014 and 2015 was that at least 1 spouse was from a 1-child family. The second most common justification for applying was remarriage.

#### 3.1.3 Age of parents applying for a second child

**Figure 3:**
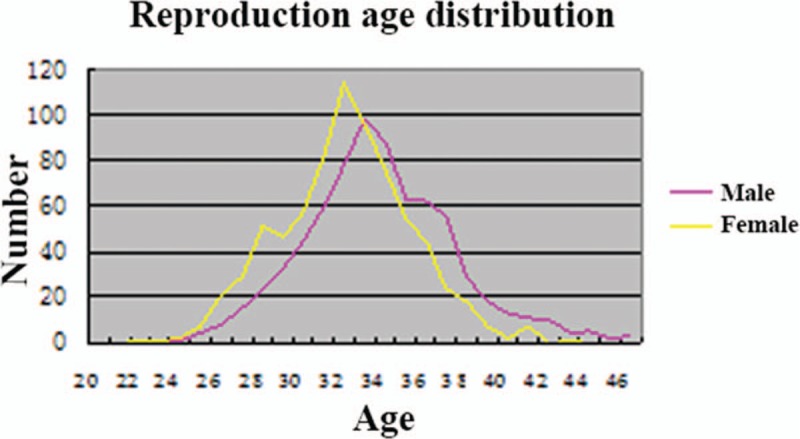
Ages of parents applying for a second child during the years of the 2-child policy (2014–2015). The mean ages of wives and husbands were 32.09 and 33.73 years, respectively.

A total of 907 families applied for a second child under the 2-child policy during the years 2014 to 2016. Of these, 52 women were lost to follow-up and 39 women failed to become pregnant. Thus, 792 women became pregnant. Until the end of this study (December 31, 2016), 739 women gave birth to a second child. The mean age of these 739 women was 32.09 years, which is older than the actual national average for second pregnancies (29.61 year) and also older than the ideal age for second pregnancies (28.40 year) in 2012 in China^[7]^ (Fig. 3 and Table 1). And the mean age of their husbands was 33.73years. The cesarean delivery rate was 59.68% (441/739) in Jiangbei district, which was higher than annual national cesarean delivery rate (54.47%) in China, 49.82% in East China, and 50.40% in Jiangshu and Zhejiang Provinces^[8,9]^ (Table 2).

**Table 1:**
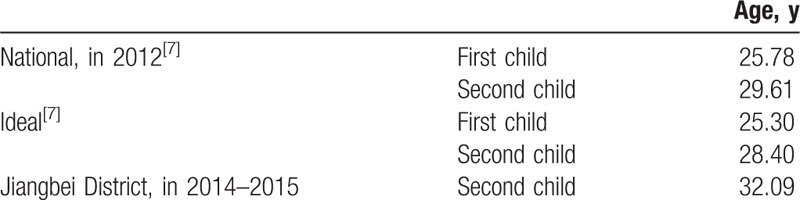
Ages of women at parturition.

**Table 2:**
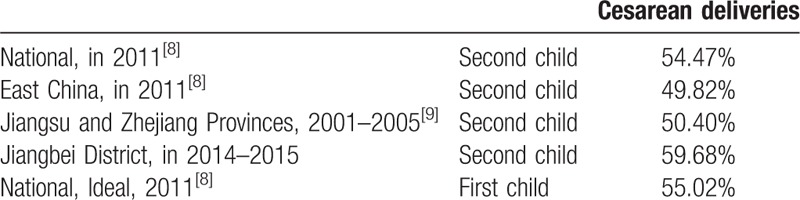
Rates of cesarean deliveries.

We further found that age of the woman was positively associated with the rate of cesarean delivery (Table 3); as the woman's age increased, the cesarean delivery rate became markedly higher than the vaginal delivery rate (*P* < .001).

**Table 3:**
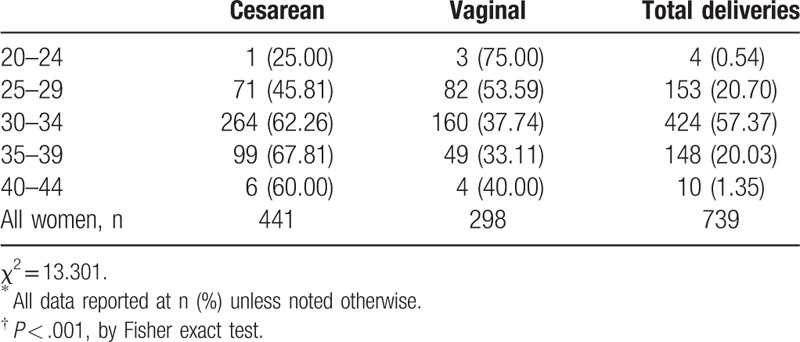
Method of delivery by women's age in years^∗^^,^^†^.

Between delivery of the first child and the second, many women had undergone artificial abortion due to an unexpected pregnancy, especially before the 2-child policy was in effect (Table 4). However, there was no association between abortion incidence and method of delivery (*P* = .31).

**Table 4:**
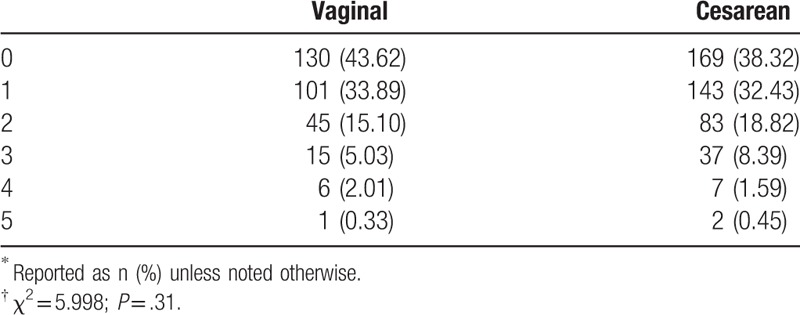
Method of delivery of the second child according to the number of previous artificial abortions^∗^^,^^†^.

### 3.2 Adverse pregnancy analysis

#### 3.2.1 Elderly parturient older parturients and prenatal screening

In 739 parturients, 158 (21.38%) were ≥35 years, among whom most were aged 35 to 39 years (93.67%), and only 10 (6.33%) were aged 40 to 44 years (Table 5). Among the 158 parturients aged ≥35 years, 98 (62.03%) received prenatal screening, in which 61 and 37 chose noninvasive DNA and amniocentesis, respectively, and 60 women (37.97%) refused prenatal screening. Among the women who allowed prenatal screening, most chose noninvasive DNA.

**Table 5:**
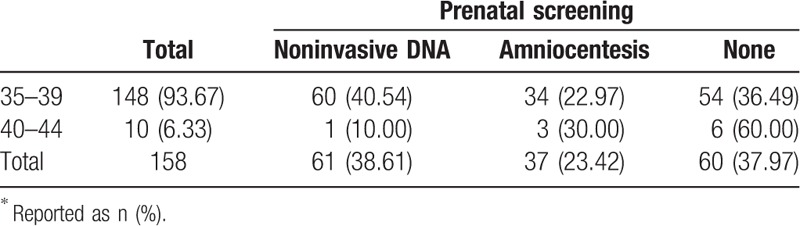
Methods of prenatal screening selected by older women stratified by years of age^∗^.

#### 3.2.2 Gestational diabetes mellitus (GDM)

Gestational diabetes mellitus (GDM) is a common complication in pregnant women (Table 6). In the 739 parturients, 105 (14.21%) were complicated with GDM. The mean age of the parturients with GDM and their husbands were 33.42 and 35.12 years, respectively. The percentage of parturients with GDM significantly increased with age at delivery (*P* < .001).

**Table 6:**
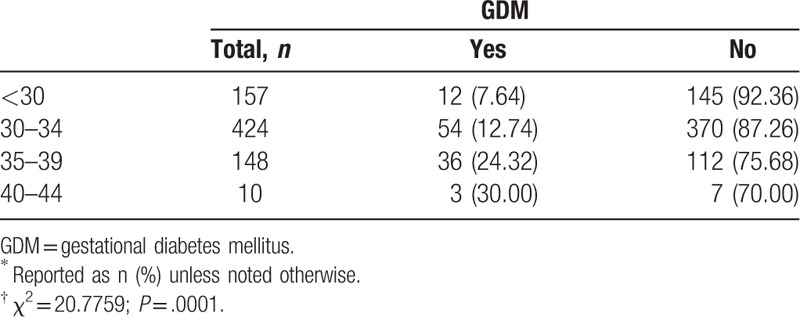
Presence of GDM according to women's age at delivery^∗^^,^^†^.

We further investigated the rates of other adverse pregnancy outcomes in the 105 parturients with GDM (Table 7) according to age (<35 or ≥35 years), and found a significant difference between these groups (*P* = .03). Specifically, compared with women with GDM aged <35 years, parturients aged ≥35 years had significantly higher rates of low birth weight infants, premature infants, and postpartum hemorrhage. In contrast, the younger group had significantly higher rates of macrosomia, thyroid dysfunction, and gestational liver damage than the older parturients (*P* < .05)

**Table 7:**
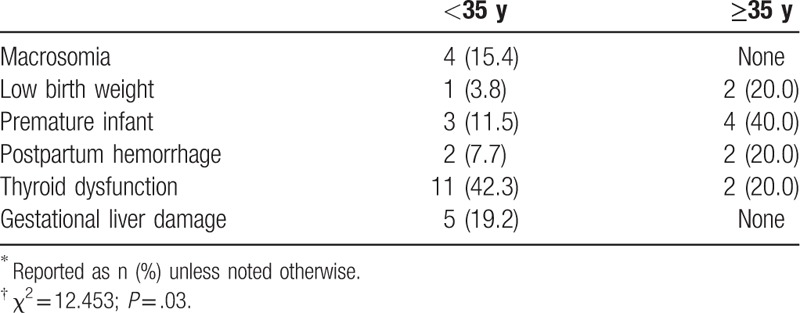
Adverse pregnancy outcomes in 105 women with GDM according to age status^∗^^,^^†^.

### 3.3 Postpartum hemorrhage

There were 33 (4.47%) who suffered postpartum hemorrhage (Table 8). The mean age of the women with postpartum hemorrhage was 33.18 years (33.18 ± 3.35), and the mean age of their husbands was 35.72 years. The percentage of women aged ≥35 years who experienced postpartum hemorrhage was higher than that of the younger group, but the difference was not significant (*P* = .06).

**Table 8:**
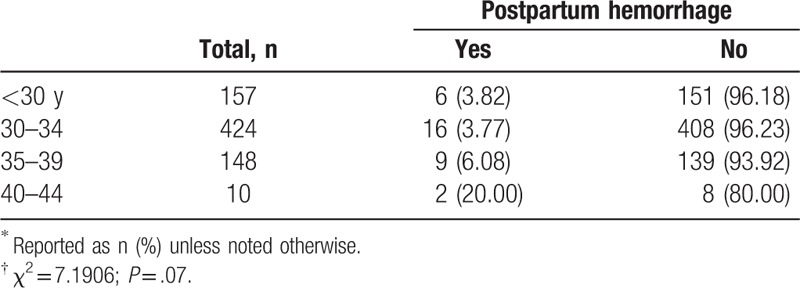
Postpartum hemorrhage according to women's age at delivery^∗^^,^^†^.

We also investigated the risk factors for postpartum hemorrhage (Table 9). Overall, there were no significant differences between the younger (<35 years) and older (≥35 years) parturients with regard to risk factors associated with postpartum hemorrhage (*P* = .15). Notably, 30 of 33 (90.9%) women with postpartum hemorrhage experienced cesarean delivery, and 25 and 30 with cesarean delivery at first and second birth, respectively.

**Table 9:**
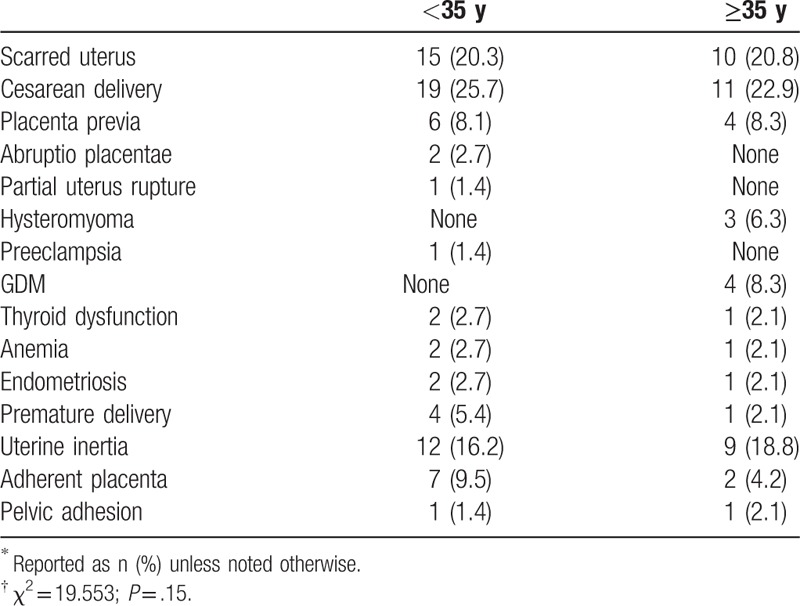
Risk factors for postpartum hemorrhage according to age status^∗^^,^^†^.

### 3.4 Neonatal adverse outcomes

We were very concerned with adverse outcomes of neonates (Table 10). Among the 739 parturients, 82 (11.10%) gave birth to 85 neonates with adverse outcomes, which included the following, in descending order of occurrence: macrosomia (birth weight ≥4.0 kg); prematurity (pregnancy less than 37 weeks); low birth weight (<2.5 kg) and extreme low birth weight (<1.5 kg); neonatal asphyxia; malformation; and neonatal jaundice.

**Table 10:**
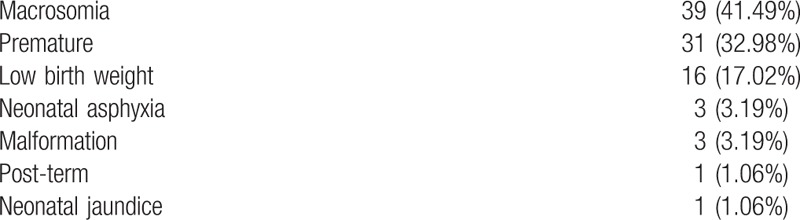
Adverse outcomes of neonates, n (%).

Some infants had combinations of adverse outcomes. Among the 39 infants with macrosomia, 4 had GDM, and one each also had polydactyly, polyhydramnios, or umbilical abnormities. Among the infants with low birth weight, there were 3 with extreme low birth weight, including 1 dead infant. Infants with malformations included one each with polydactyly, syndactylia, and atrial septal defect.

### 3.5 Pathological termination of pregnancy

Among the 907 women applying for a second child, 39 pregnancies were terminated for various reasons. There were 28, 10, and 1 pregnancies that were terminated during early, middle, and late gestation, respectively, where early, middle, and late gestation was defined as <12 weeks, weeks 12 to 28, and >28 weeks (Table 11). The rates of terminations between the young (<35 years) and older (≥35 years) parturients were statistically similar.

**Table 11:**
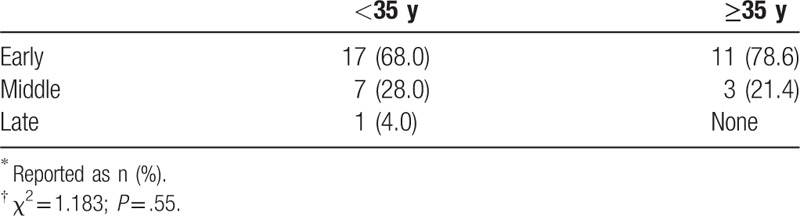
Pregnancy terminations at early, middle, and late gestation according to maternal age^∗^^,^^†^.

Because most pregnancies terminated during early gestation, we selectively analyzed the reasons for termination during this period (Table 12). The rates for early terminations were similar between the 2 age groups (*P* = .49).

**Table 12:**
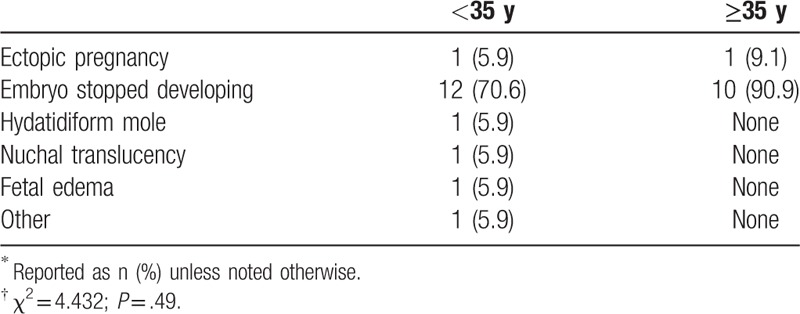
Reasons for pregnancy terminations in early gestation, by maternal age^∗^^,^^†^.

### 3.6 The reason for loss

#### 3.6.1 Loss to follow-up

In this study, 907 women applied for a second birth, among whom 52 women (5.73%) were finally lost to follow-up, mostly due to lose of contact. In addition, some households changed their contact information and migrated without informing us.

## 4 Discussion

This study was conducted to facilitate foreseeing the issues that may affect obstetrical departments, related to the introduction of the universal 2-child policy in 2016. We retrospectively investigated the demographics and health-related outcomes of second pregnancies in families who applied for a second child in Jiangbei District of Ningbo City during the years of the 2-child policy, 2014 to 2016. We found that the number of applications for a second child increased after the policy went into effect in 2014, with a total of 907 families applying during 2014 to 2016. Until December 31, 2016, 739 women gave birth to their second child, among whom 21.38% were 35 years of age or older. Rates of cesarean deliveries (59.68%) and GDM (14.21% of women) were each positively associated with the age of the mother (*P* < .001, each).

After the 2-child policy was implemented in 2014, the number of families applying for a second birth in Jiangbei district doubled, relative to the number before 2014. Within the first 2 months, the number of applications quickly increased and then decreased to a stable high level in 2014. The predominant justification for applying for a second birth was a spouse from a 1-child family. Because the 1-child family planning policy had been implemented for more than 30 years, the desire to produce a second child in most families had been repressed. We forecast that, with the 2-child policy in place, an increase in the reproductive population will steadily increase in the long term.

In our study, substantial numbers of women applying for a second birth were aged 35 years or more, because their desire for a second child had been prohibited during the period of the 1-child policy. These older parturients often had a history of accidental pregnancy and subsequent abortion. We can conclude that the desire of older women for a second child will persist for a long time in China, with the introduction of the new universal 2-child policy.

In Jiangbei district, the mean age of parturients at the second birth was 32.09 years and their husband's mean age was 33.73 years, which are both older than that of several other provinces in previous reports.^[7]^ Of particular note, 30% to 45% of women with adverse pregnancy outcomes were older parturients (≥35 years), especially those with adverse outcomes in early gestation. The actual percentage of older parturients with an adverse pregnancy outcome was probably higher, because most families apply for a second birth after the pregnancy has been confirmed, and if termination occurs due to early dysembryoplasia, the application for a second child is not made. It may be that adverse pregnancy outcomes are more likely in older parturients, who are more likely to have decreased egg quality, embryonic chromosomal mutations, and embryo implantation issues caused by an injured uterus. Several studies have shown that embryos in older pregnant women are more prone to chromosomal abnormality, which may be due to a decrease in ovarian reserve function and follicular mass, or spindle aging caused by failure of chromosomal segregation that leads to an increase in trisomy.^[10,11]^

We found that fetal malformation was the major reason for pregnancy termination during the second trimester. However, there was no significant difference in the rate of fetal malformation between the 2 age groups (<35 and ≥35 years), perhaps due to the low efficiency in screening for Down syndrome during the first and second trimester, or because the fertilized egg in older women is more susceptible to a fatal malformation that results in early abortion. On the contrary, older women are more prone to adverse pregnancy issues associated with increased age (e.g., sudden stillbirth, pregnancy complications, and cervical incompetence), and also scarring of the cervical and endometrial cavity due to repeated surgeries, which are major reasons for termination during the second trimester.

Regarding prenatal screening, invasive methods such as chorionic villus sampling and amniocentesis are painful and stressful^[12]^ and pregnant women therefore prefer noninvasive prenatal testing (NIPT).^[13]^ However, cell-free circulating fetal DNA NIPT is limited by high cost, inaccurate results, and risk assessments that are mostly confined to trisomies 21, 18, and 13.^[14–16]^ In the present study, more than one-third of the older women refused all prenatal testing. Yet, if prenatal testing is applied after 10 weeks of gestation and a probable adverse outcome is detected, termination during the second trimester is more likely to affect the woman's health and future fertility. Therefore, it is essential to develop a better method of NIPT for older pregnant women.

Older pregnant women are also prone to health complications. In our study, the rate of GDM was 14.21%, which is higher than the average range of 1% to 5% in China, and the rate is positively associated with age. This is consistent with the study by Balasch and Gratocos.^[17]^ In older pregnant women, the rate of insulin utilization, postpartum hemorrhage, and cesarean section is significantly higher. Thus, individualized diagnosis of GDM and its management should fully consider the patient's age.^[18]^

Another important problem of older pregnant women is the high rate of cesarean section delivery. The major reasons for cesarean section in older women include a previous cesarean section, and pathological and psychological factors.^[19]^ In addition, during subsequent pregnancies, women with a history of cesarean section are at a greater risk than women with an intact uterus of the following adverse events: cesarean scar pregnancy, placenta previa, uterine rupture, postpartum hemorrhage, intrapartum hysterectomy, and preterm birth.

In the present study, we also found that the rate of postpartum hemorrhage in older women was slightly higher than the younger group, and in most cases was combined with hysteromyoma, GDM, pelvic adhesion, anemia, and thyroid disease. These combinations make the complications of pregnancy more intractable and complex to treat.

In conclusion, this retrospective cohort survey assessed the health-related pregnancy issues and outcomes in families who applied for a second child in Jiangbei District of Ningbo City within 2 years of introduction of China's 2-child policy. The results showed that the number of second pregnancies increased, and there are important issues related to this increase that must be addressed by obstetric departments, with appropriate adjustments to perinatal health care schemes. We recommend, first, that women who apply for a second child should undergo early comprehensive health and fertility assessments. Second, fast and accurate minimally invasive prenatal screening methods must be developed and made available to pregnant women. Third, pregnancy complications must be monitored, especially during the last trimester, and especially in older pregnant women. Finally, long-term adjustments must be made to the perinatal management system to meet the needs of greater numbers of women with second pregnancies.

### 4.1 Limitations of this study

The size of samples was small because the samples were only from 1 district in Ningbo City. For some complications, the number of samples did not meet the requirement of statistical analysis. In further study, we need to collect a large size of samples from more districts to investigate the new challenges related to 2-child policy in China.

## Author contributions

**Conceptualization:** Jun Fu.

**Data curation:** Jun Fu, Fei Ji, Huijuan Li.

**Formal analysis:** Yu Qu.

**Funding acquisition:** Jun Fu.

**Investigation:** Huijuan Li, Fangyuan Chen.

**Methodology:** Fei Ji.

**Project administration:** Jun Fu, Yu Qu.

**Resources:** Fangyuan Chen.

**Software:** Fangyuan Chen.

**Supervision:** Jun Fu, Yu Qu, Huijuan Li.

**Validation:** Fei Ji.

**Visualization:** Fangyuan Chen.

**Writing – original draft:** Jun Fu.

**Writing – review & editing:** Jun Fu.
